# Microvascular obstruction and endothelial activation are independently associated with the clinical manifestations of severe falciparum malaria in adults: an observational study

**DOI:** 10.1186/s12916-015-0365-9

**Published:** 2015-05-27

**Authors:** Josh Hanson, Sue J. Lee, Md Amir Hossain, Nicholas M. Anstey, Prakaykaew Charunwatthana, Richard J. Maude, Hugh W. F. Kingston, Saroj K. Mishra, Sanjib Mohanty, Katherine Plewes, Kim Piera, Mahtab U. Hassan, Aniruddha Ghose, M. Abul Faiz, Nicholas J. White, Nicholas P. J. Day, Arjen M. Dondorp

**Affiliations:** Mahidol-Oxford Tropical Medicine Research Unit, Faculty of Tropical Medicine, Mahidol University, Bangkok, Thailand; Global Health Division, Menzies School of Health Research, Darwin, Australia; Centre for Tropical Medicine, Nuffield Department of Clinical Medicine, University of Oxford, Oxford, UK; Department of Medicine, Chittagong Medical College Hospital, Chittagong, Bangladesh; Department of Medicine, Ispat Hospital, Rourkela, Orissa India; Centre for Specialized Care and Research, Chittagong, Bangladesh; Dev Care Foundation, Dhaka, Bangladesh

**Keywords:** Falciparum malaria, Microcirculation, Pathophysiology, Endothelial dysfunction

## Abstract

**Background:**

Microvascular obstruction and endothelial dysfunction have both been linked to tissue hypoperfusion in falciparum malaria, but their relative contributions to the disease’s pathogenesis and outcome are unknown.

**Methods:**

Microvascular blood flow was quantified in adults with severe falciparum malaria on their admission to hospital; plasma biomarkers of endothelial function were measured simultaneously. The relationship between these indices and the patients’ clinical findings and in-hospital course was examined.

**Results:**

Microvascular obstruction was observed in 119/142 (84 %) patients; a median (interquartile range (IQR)) of 14.9 % (6.6–34.9 %) of capillaries were obstructed in patients that died versus 8.3 % (1.7–26.6 %) in survivors (*P* = 0.039). The proportion of obstructed capillaries correlated with the estimated parasite biomass (r_s_ = 0.25, *P* = 0.004) and with plasma lactate (r_s_ = 0.38, *P* <0.0001), the strongest predictor of death in the series. Plasma angiopoietin-2 (Ang-2) concentrations were markedly elevated suggesting widespread endothelial activation; the median (IQR) Ang-2 concentration was 21.9 ng/mL (13.4–29.4 ng/mL) in patients that died versus 14.9 ng/mL (9.8–29.3 ng/mL) in survivors (*P* = 0.035). Ang-2 concentrations correlated with estimated parasite biomass (r_s_ = 0.35, *P* <0.001) and plasma lactate (r_s_ = 0.37, *P* <0.0001). Microvascular obstruction and Ang-2 concentrations were not significantly correlated with each other (r_s_ = 0.17, *P* = 0.06), but were independently associated with plasma lactate (*P* <0.001 and *P* = 0.002, respectively).

**Conclusions:**

Microvascular obstruction and systemic endothelial activation are independently associated with plasma lactate, the strongest predictor of death in adults with falciparum malaria. This supports the hypothesis that the two processes make an independent contribution to the pathogenesis and clinical manifestations of the disease.

## Background

In 1892, five years before Ross identified mosquitoes as the vector, Marchiafava and Bignami explored the clinical presentations and related pathology of malaria in their seminal monograph, *On Summer-Autumnal Fevers* [1]. They observed that only *Plasmodium falciparum* caused ‘malignant malaria’ and presented a post-mortem series that identified the sequestration of parasitized red blood cells (pRBCs) in the microcirculation as the pathological signature of the disease. They highlighted the relationship between the extent of this sequestration and the patients’ clinical course, hypothesizing that the resulting microvascular obstruction was responsible for many of falciparum malaria’s distinctive clinical manifestations [1]. In the subsequent 120 years, post-mortem series have validated their findings, confirming microvascular obstruction’s central role in the disease’s pathogenesis [2–5].

More recently the potential pathological contributions of systemic endothelial activation and dysfunction have been recognized [6, 7]. These processes result, in part, from reduced nitric oxide bioavailability and may further impair microvascular blood flow [8]. Plasma concentrations of a key autocrine mediator of endothelial activation, angiopoietin-2 (Ang-2), correlate with later death in falciparum malaria in both adults [7, 9] and children [10]. Endothelial activation potentiates sequestration through the upregulation of endothelial ligands [5]; however, endothelial activation occurs in both mild and severe falciparum malaria [11] and in many other infectious and non-infectious conditions [12, 13]. And while endothelial activation in severe malaria is associated with death independent of total parasite biomass [7], its association with disease severity and mortality independent of direct measures of microvascular obstruction has never been evaluated.

This study explored the relationship between microvascular obstruction (assessed directly with orthogonal polarization spectral (OPS) imaging) and endothelial activation and function (quantified with plasma biomarkers) in adults with severe falciparum malaria to determine their association with the disease’s clinical manifestations and outcome.

## Methods

Clinical, laboratory and OPS imaging data were gathered prospectively from three groups of adult patients enrolled in studies of severe falciparum malaria between April 2003 and August 2011. OPS imaging data from the first two groups have been presented previously: the first group (n = 43) comprised patients enrolled in a study of OPS imaging in falciparum malaria [14] and the second group (n = 26) comprised patients enrolled in a study assessing the fluid management of patients with severe falciparum malaria [15]. The third group (n = 91) comprised patients enrolled in studies examining adjunctive therapies in falciparum malaria and pathophysiology [16, 17]. Their OPS imaging data was analysed specifically for this series.

All patients were hospitalized at Chittagong Medical College Hospital, Bangladesh or Ispat General Hospital, Rourkela, India. Malaria transmission is seasonal at both sites. Falciparum malaria was diagnosed if asexual forms of *P. falciparum* were present on a blood film. When expert microscopy was not available immediately, patients were enrolled if an immunochromatographic rapid diagnostic test (Paracheck Pf, Orchid Biomedical Systems, Goa, India) was positive, and *P. falciparum* infection was confirmed later by microscopy of a simultaneously collected blood slide.

All patients satisfied a strict definition of severe falciparum malaria. Patients had at least one of the following modified World Health Organization (WHO) criteria: cerebral malaria (Glasgow Coma Scale (GCS) <11); severe anaemia (haematocrit <20 % with a parasite count >100,000/mm^3^); renal failure (blood urea nitrogen >21.4 mmol/L or serum creatinine level >265 μmol/L); pulmonary oedema (oxygen saturation <90 % and bibasal crepitations); generalized convulsions; acidosis (venous bicarbonate <15 mmol/l); hyperparasitaemia (peripheral parasitaemia >10 %); hyperlactataemia (venous lactate >4 mmol/L); jaundice (bilirubin >42.8 mmol/L and a parasite count >100,000/mm^3^); hypoglycaemia (glucose <2.2 mmol/L); and spontaneous bleeding or shock (systolic blood pressure <80 mmHg with cool extremities). Multi-organ dysfunction was defined as the presence of more than one of these criteria (excluding hyperparasitaemia), although in this definition coma and seizures were classified as one criterion, as were acidosis and hyperlactatemia; if data were missing, the patient was presumed not to satisfy that criterion. Patients were excluded if they were <13 years of age or if they had received parenteral antimalarial treatment for >24 h before enrolment.

On enrolment, a standardized history was taken, a physical examination performed and venous blood collected. Nine patients in the first group were treated with intravenous quinine but all the remaining patients received intravenous artesunate. Patients received standard supportive care as per contemporary WHO treatment guidelines [18–20], although the availability of supportive therapies including mechanical ventilation and renal replacement therapy (RRT) was limited, particularly in the earlier study years.

The absolute peripheral parasite count (parasites/μl) was calculated on admission from the thin blood film using the formula: parasite count/1,000 RBC × 125.6 × haematocrit (%) or from the thick film using the formula: parasite count/200 white blood cells × 40. Laboratory indices were measured at field sites using a portable handheld analyser (i-Stat®, Abbott, Princeton, NJ, USA). For other indices plasma samples were processed and stored at −80 °C for further analysis in Bangkok, Thailand and Darwin, Australia. Plasma *Plasmodium falciparum* histidine-rich protein 2 (*Pf*HRP2) was measured using ELISA (Cellabs, Sydney, New South Wales, Australia), according to the manufacturer’s instructions with minor modifications as described previously [21]. Ang-2 and vascular endothelial growth factor (VEGF) were also measured using ELISA (R&D Systems, Minneapolis, MN, USA). L-arginine, the substrate for endothelial cell NO synthase, and asymmetric dimethylarginine (ADMA), an endogenous inhibitor of NO synthase, were measured using high performance liquid chromatography as described previously [22]. As there are no published reference ranges for these measures of endothelial function in the normal Bangladeshi population, samples were collected - and a reference range determined - from 45 healthy volunteers. It was not possible to export Indian plasma and so the 15 Indian patients did not have their Ang-2, L-arginine, VEGF or ADMA measured.

### OPS recording and analysis

Video recordings of the microcirculation in the rectal mucosa were collected at the time of study enrolment, with an OPS device (either Cytoscan® from Cytometrics, Heathpark, Honiton, Devon, UK or Microscan® from Microvision Medical, Amsterdam, The Netherlands), which was applied gently to prevent pressure artefact. The video recordings were imported into Final Cut Pro (version 3.0.2, Apple, Cupertino, CA, USA), and were analysed using image analysis software (OpenLab 3.1.5, Improvision, Waltham, MA, USA). Separate recordings of at least 10 seconds were collected at three different sites on the rectal mucosa of all patients. Blood flow was measured by tracing the movement of individual erythrocytes over time; only erythrocytes that could be tracked over three successive frames were assessed. Obstructed microvascular blood flow was defined as a blood flow velocity <100 μm/s. Assessment was performed on three occasions: two reviewers (JH and PC) separately analysed the recordings from the first group blinded to patient details and outcome in 2006. In this group of patients a mean of 50 (95 % confidence interval (CI) 37–62) capillaries were assessed. In 2009 the recordings from the second group were analysed by the clinician (JH) who had managed the patients a year previously (although patients were identified only by a patient code and the results were validated by blinded reviewers (RJM and HK) [15]). In 2013 the third group was analysed by a single reviewer (JH) blinded to patient details and outcome. In the second and third group of patients the blood flow in 60 capillaries was assessed in all patients.

### Statistics

Data were collected and analysed using statistical software (Stata version 10, StataCorp, College Station, TX, USA). Associations between continuous variables were examined using Spearman’s rank correlation coefficient. Groups were compared using the Kruskal-Wallis test, the chi-squared and Fisher’s exact test. The strongest predictor of death was identified using backward stepwise estimation in a logistic regression model with random effects for study year. All risk factors that were significant in the univariate analysis at *P* <0.05 were included in the initial model (Table 1). Only variables that were significant at *P* <0.05 were retained in the final model.

**Table 1:** Baseline characteristics of the patients and association with outcome

Variable	N	All n = 142	Survivors n = 95	Deaths n = 47	*P* ^b^
Age (years)	141	35 (25–45)	31 (24–46)	35 (25–45)	1.00
Sex (% male)	141	71 %	74 %	64 %	0.24
Glasgow Coma Score	142	9 (6–13)	10 (8–14)	8 (4–10)	<0.001
Mean arterial pressure (mmHg)	140	82 (72–93)	80 (72–90)	83 (71–97)	0.32
Respiratory rate (breaths/min)	137	30 (26–36)	30 (25–36)	32 (28–40)	0.02
Oxygen saturation (%)	138	96 (94–97)	96 (94–97)	96 (93–97)	0.39
Temperature (°C)	142	38 (37.2– 38.9)	37.9 (37.2–38.6)	38.2 (37.1–39.2)	0.25
Heart rate (beats/min)	138	109 (96–122)	107 (93–119)	117 (102–132)	0.02
Plasma sodium (mmol/L)	140	135 (132–139)	135 (132–139)	135 (132–140)	0.4
Plasma potassium (mmol/L)	139	4 (3.6–4.5)	3.9 (3.6–4.4)	4.2 (3.8–4.6)	0.05
Blood urea nitrogen (mmol/L)	141	14.3 (8.8–24.1)	12.9 (8.2 –22)	17.9 (10.3–29.3)	0.02
Plasma creatinine (μmol/L)	140	136 (88–220)	122 (86–193)	150 (97–300)	0.16
Plasma lactate (mmol/L)	141	4.3 (2.6–6.6)	3.7 (2.2–5.1)	6.3 (4.1–10)	<0.001
Base deficit (meq/L)	140	8 (3–12)	6.5 (2–10)	12 (5–17)	<0.001
White cell count (x 10^9^/L)	104	8.5 (6.1–10.9)	8 (5.4–10)	10.9 (7.2–11.5)	0.002
Haemoglobin (g/dL)	139	9.6 (7.8–11.3)	9.3 (8–11.2)	10.2 (7–12)	0.64
Platelet count (x 10^9^/L)	107	36 (21–66)	43 (24–87)	27 (15–41)	0.02
Microvascular obstruction (%)^a^	142	11.9 (3–26.7)	8.3 (1.7–26.6)	14.9 (6.6–34.9)	0.039
*Pf*HRP2 (ng/mL)	135	2,268 (960–4,789)	2,151 (881–4,261)	2,584 (1,075–5,946)	0.17
Parasite count (parasites/uL)	134	67,133 (20,253–274,499)	97,968 (11,681–310,546)	60,288 (24,116–176,845)	0.59
Angiopoietin-2 (ng/mL)	126	19.1 (10.8–29.1)	14.9 (9.8–29.3)	21.9 (13.4–29.4)	0.035
L-arginine (μmol/L)	126	59.6 (44.3–79.9)	59.6 (44.1–77.8)	60.1 (38.3–71.5)	0.78
ADMA (μmol/L)	126	0.77 (0.58–1.01)	0.77 (0.57–0.99)	0.77 (0.58–1.18)	0.58
L-arginine: ADMA ratio	126	73.3 (59.8–90.6)	72.7 (62.7–89.6)	73.5 (58.6–90.7)	0.43
VEGF (pg/mL)	125	27.3 (0–59.2)	24.9 (0–54.7)	34.1.7 (0–64.9)	0.93
Plasma glucose (mmol/L)	137	6.6 (5–9.2)	6.8 (5.3–9.3)	6.2 (3.6–9.1)	0.07

All values represent the median (interquartile range) unless otherwise stated. ^a^Determined using orthogonal polarization spectral imaging. The percentage of capillaries with a blood flow of <100 μm/s. ^b^Statistical significance for a difference between survivors and non-survivors. ADMA: Asymmetric dimethylarginine; *Pf*HRP2*: Plasmodium falciparum* histidine-rich protein 2; VEGF: Vascular endothelial growth factor

Logistic regression models were used to quantify the odds of death with study and year fitted as a random effect to account for clustering and, in the case of the OPS imaging data, any unobserved heterogeneity due to inter-rater variability. An interaction term for plasma Ang-2 concentration and microvascular obstruction was created to determine whether their association with disease severity and death could be interpreted without effect modification.

### Ethics review

All of the studies from which data were analysed received prospective ethical approval from the Bangladeshi Medical Research Council, the institutional ethical board of Ispat General Hospital and the Oxford Tropical Medicine Research Ethical Committee. ISRCTN registration numbers: ISRCTN 27232551, ISRCTN 20156397 and ClinicalTrials.gov identifier NCT00692627. Family members provided written informed consent, via a local interpreter, before participants were enrolled into the studies.

## Results

### Patients

**Fig. 1:**
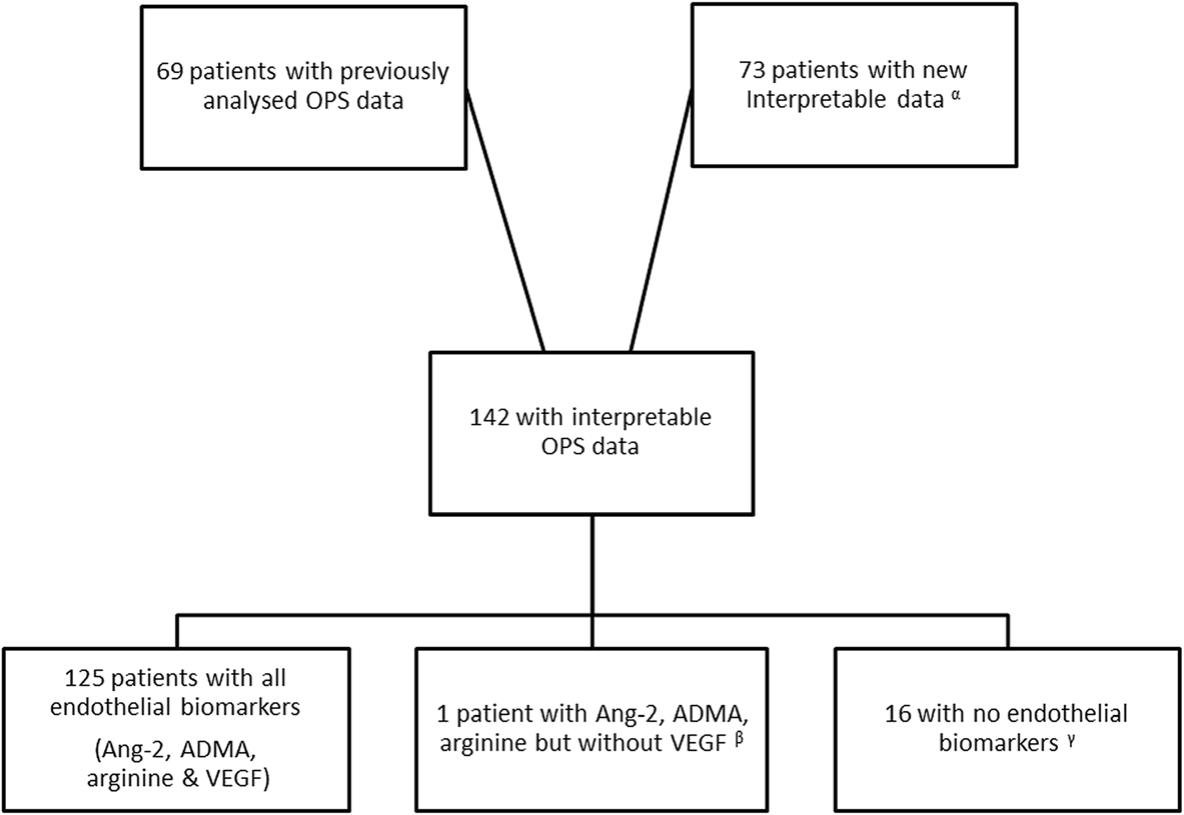
Selection of OPS imaging data for analysis. ^α^There were 91 new patients with OPS imaging data for analysis. Eighteen of these patients had poor quality images that precluded analysis: six had pressure artefact, three had a recording that was too short and poorly located, three had a recording that was too short, two had a recording that was short and had pressure artefact, one had unfocussed images, one was poorly located, one had pressure artefact and was poorly located, and one was short, had pressure artefact and was poorly located. These eighteen patients were excluded from the analysis. ^β^One patient had insufficient specimen for a VEGF determination. ^γ^One Bangladeshi patient had no admission specimen; the 15 Indian patients could not have plasma exported. ADMA: Asymmetric dimethylarginine; Ang-2: Angiopoietin-2; OPS: Orthogonal polarization spectral; VEGF: Vascular endothelial growth factor

OPS imaging data were available for 160 patients (69 previously reported [14, 15] and 91 new patients). Poor quality images precluded reliable assessment of 18 of the new patients’ films (Fig. 1), leaving a total of 142 patients’ recordings for analysis (Table 1). Overall 47 (33 %) of these 142 patients died despite the prompt use of parenteral anti-malarial therapy and management by dedicated study clinicians. Patients presented late in the course of their illness: the median (interquartile range (IQR)) duration of symptoms was 7 (5–8) days by which stage most (n = 90 (63.4 %)) had multi-organ dysfunction. Patients with multi-organ dysfunction on admission were more likely to die (39/90 (43 %)) than those with single organ involvement (8/52 (15 %), *P* = 0.001). Death also varied by study year (range 8.3–60 %, *P* = 0.02, degrees of freedom = 8). The highest case fatality rates occurred in the first two study years (60 % and 52.3 %, respectively) when few patients had access to RRT and mechanical ventilation. In the ensuing years, when this support was more accessible, the median (IQR) case fatality rate was 27.9 % (12.8–36.1). However, using a stepwise model, admission plasma lactate was identified as the strongest predictor of death (adjusted odds ratio (AOR): 1.39, 95 % CI 1.15–1.68). When controlled for disease severity (plasma lactate on admission), the association between death and multi-organ involvement and death and study year was no longer significant (*P* = 0.07 and *P* = 0.06, respectively).

### Microcirculation

**Fig. 2:**
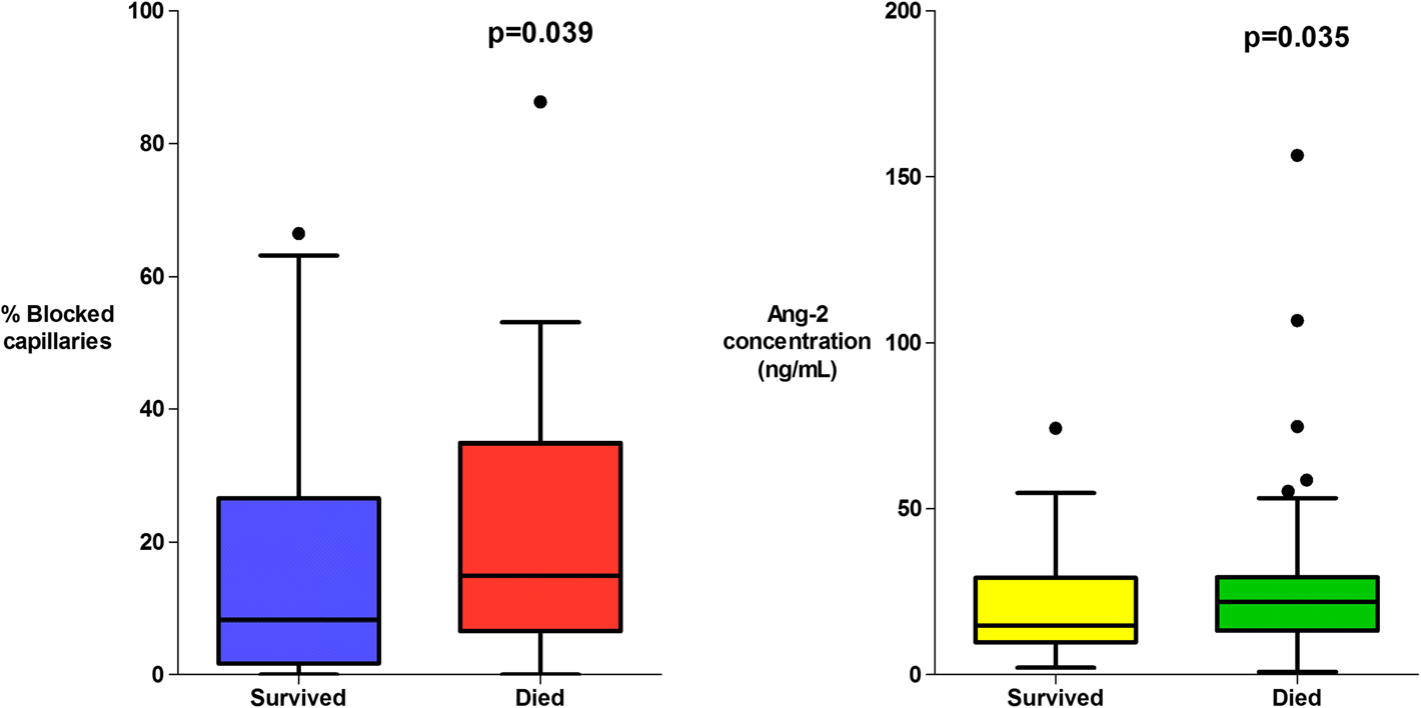
Association between microvascular obstruction, endothelial activation and patient outcome

On admission, 119/142 (84 %) patients had obstructed microvascular flow visualized with OPS imaging. The median (IQR) percentage of obstructed capillaries was greater in the patients who died (14.9 % (6.6–34.9)) than in survivors (8.3 % (1.7–26.6)) (*P* = 0.039) (Fig. 2). The relationship remained significant when controlled for inter-rater variability (AOR: 1.03, 95 % CI 1.00–1.05, *P* = 0.026) and study year (AOR: 1.03, 95 % CI 1.00–1.05, *P* = 0.027).

**Fig. 3:**
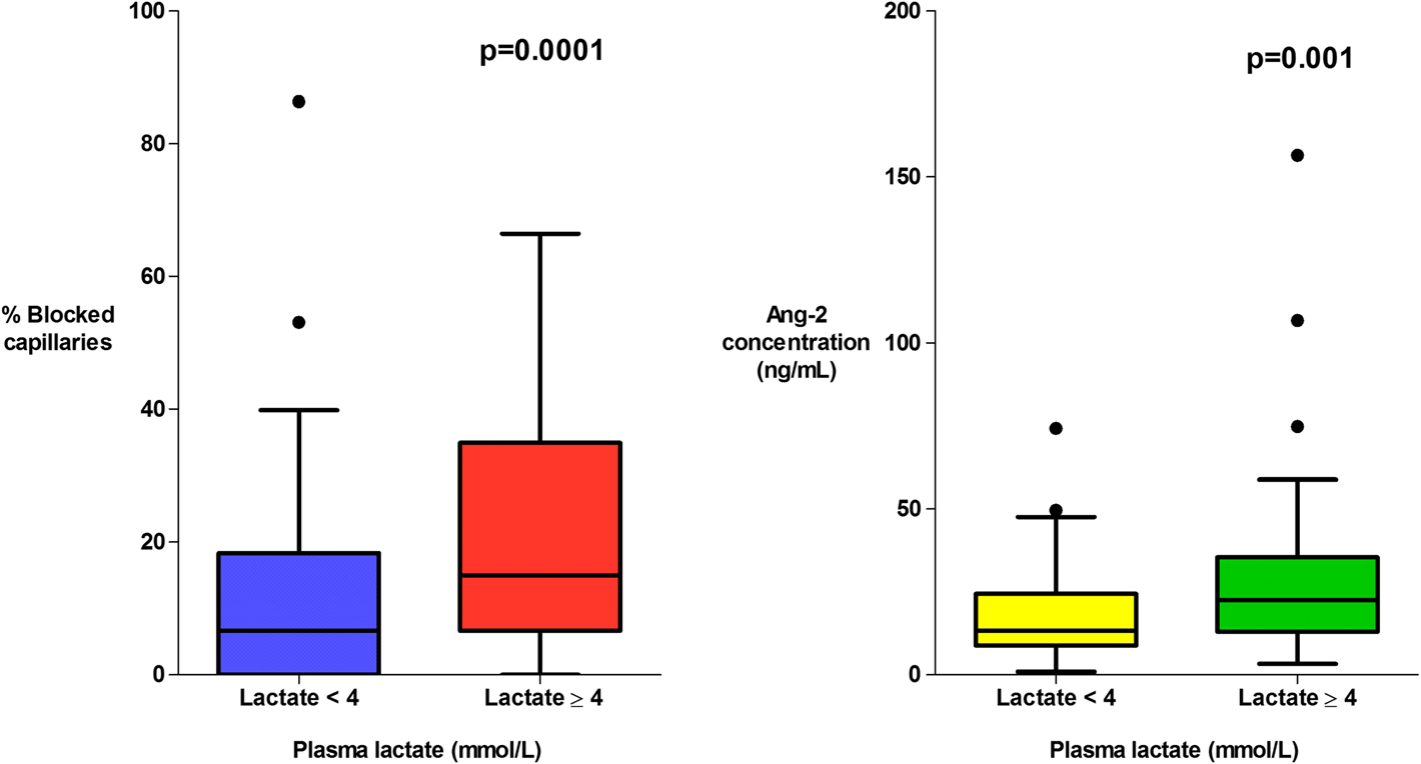
Association between microvascular obstruction, endothelial activation and plasma lactate

**Fig. 4:**
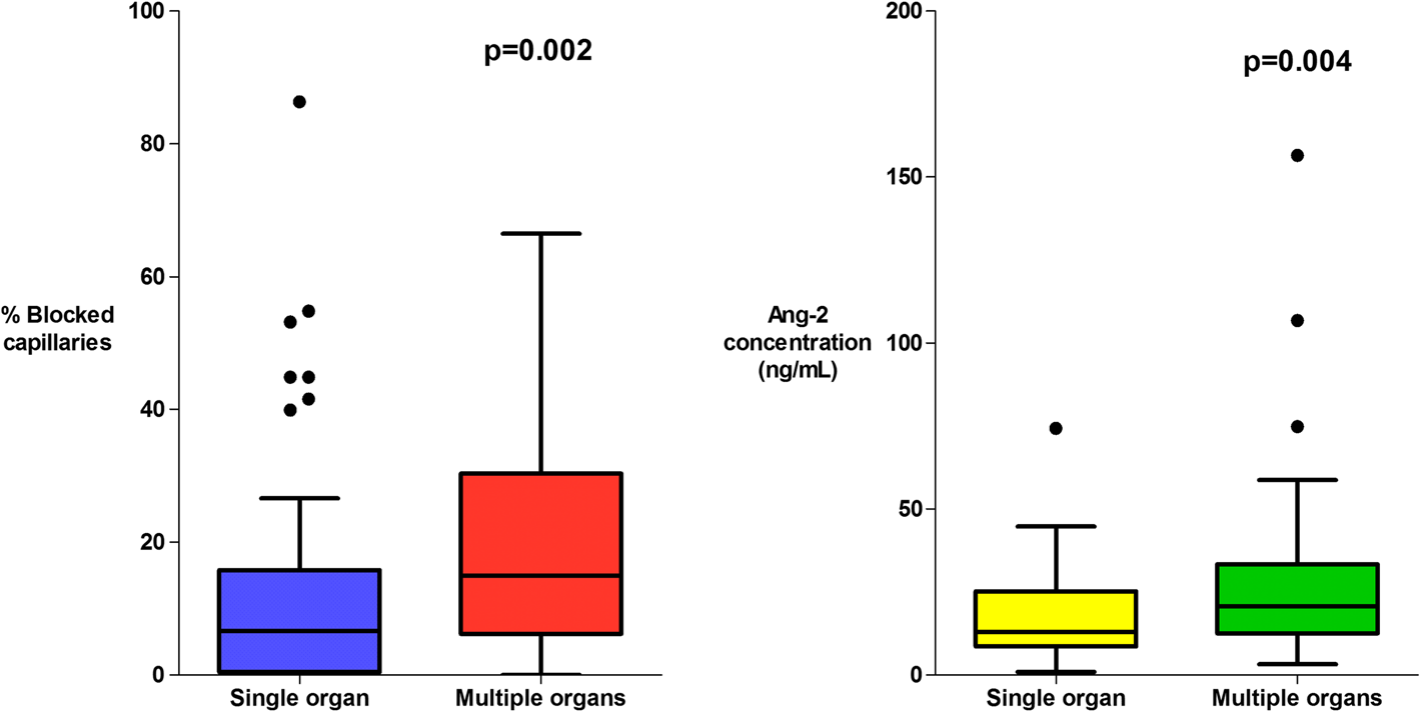
Association between microvascular obstruction, endothelial activation and multi-organ dysfunction

**Fig. 5:**
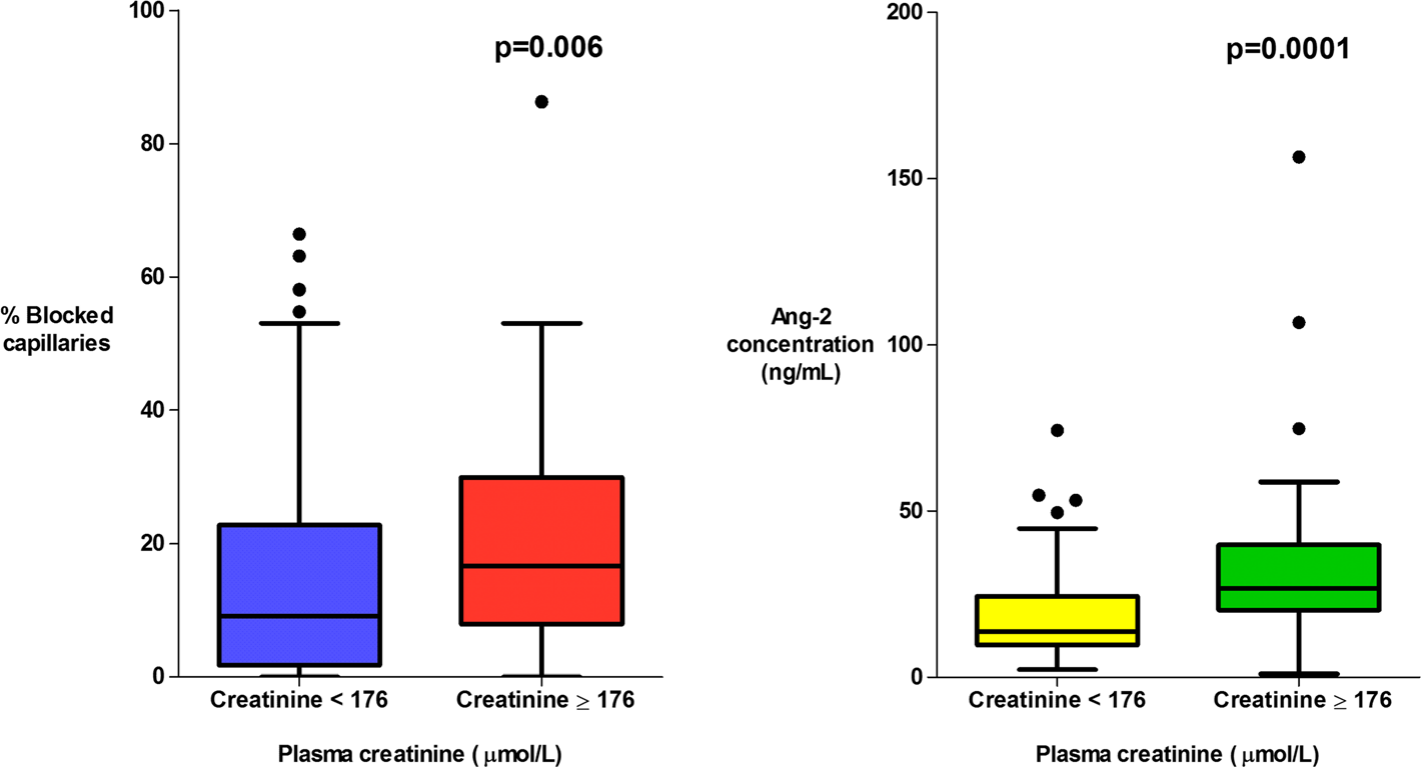
Association between microvascular obstruction, endothelial activation and acute kidney injury

**Fig. 6:**
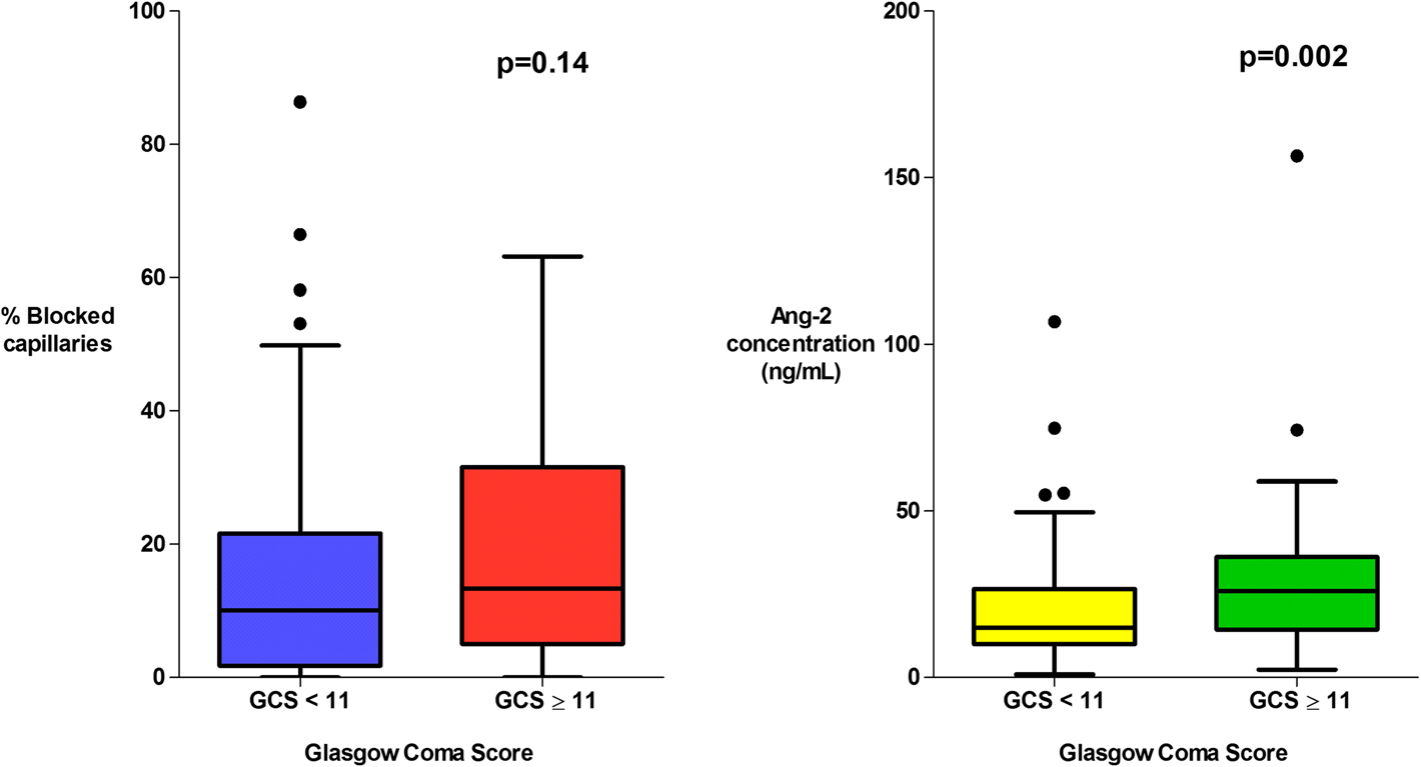
Association between microvascular obstruction, endothelial activation and coma

There was a higher proportion of obstructed capillaries in patients with a significantly elevated plasma lactate (≥4 mmol/L, the WHO cut-off for elevated lactate) (*P* = 0.0001) (Fig. 3), in patients with multi-organ dysfunction (*P* = 0.002) (Fig. 4) and in patients with acute kidney injury ((AKI), plasma creatinine ≥176 μmol/L) (*P* = 0.006) (Fig. 5). The percentage of obstructed capillaries on admission correlated with the estimated parasite biomass (r_s_ = 0.25, *P* = 0.004), and more weakly with peripheral parasite count (r_s_ = 0.19, *P* = 0.027). There was no significant relationship between the rectal OPS findings and the presence of coma (GCS <11) (*P* = 0.14) (Fig. 6). There was no statistically significant association between microvascular obstruction and plasma Ang-2 concentrations (r_s_ = 0.17, *P* = 0.057), or any of the other markers of endothelial function.

### Markers of endothelial activation and dysfunction

There was marked derangement of all the measures of endothelial activation and function on admission. Plasma Ang-2 concentrations were higher in the patients with severe malaria (median (IQR) 19.1 (10.8–29.1) ng/mL) than in healthy controls (median (IQR) 1.6 (1.4–2.3) ng/mL) (*P* = 0.0001). Admission plasma Ang-2 concentrations were significantly greater in patients who died (median (IQR) 21.9 (13.4–29.4) ng/mL) than in survivors (median (IQR) 14.9 (9.8–29.3) ng/mL) (*P* = 0.035) and were correlated with the presence of an elevated plasma lactate (*P* = 0.001) (Fig. 3), multi-organ dysfunction (*P* = 0.004) (Fig. 4), AKI (*P* = 0.0001) (Fig. 5) and estimated parasite biomass (r_s_ = 0.35, *P* <0.001). Admission plasma Ang-2 concentrations were significantly lower in the patients with coma on admission than in the non-comatose patients (*P* = 0.002) (Fig. 6).

Admission plasma ADMA concentrations were elevated in the majority of patients (median (IQR) 0.75 (0.59–1.03) μmol/L versus 0.55 (0.49–0.59) μmol/L in healthy controls (*P* = 0.0001)), and correlated with Ang-2 (r_s_ = 0.41, *P* <0.001) and plasma lactate (r_s_ = 0.22, *P* = 0.01), but not death (*P* = 0.31). Plasma L-arginine concentrations were decreased on admission in the majority of patients (median (IQR) 59.6 (44.3–79.9) pg/mL in patients versus 141 (116.6–161.6) pg/mL in healthy controls (*P* = 0.0001)), and correlated with Ang-2 (r_s_ = 0.31, *P* <0.001) and plasma lactate (r_s_ = 0.29, *P* = 0.001), but not death (*P* = 0.56). Median admission plasma VEGF concentrations were generally decreased (median (IQR) 27.3 (0–59.2) pg/mL versus 79.2 (44.0–114.0) pg/mL in healthy controls (*P* = 0.001)), and were inversely correlated with Ang-2 (r_s_ = −0.37, *P* <0.001), parasite biomass (r_s_ = −0.31, *P* <0.001) and plasma lactate (r_s_ = −0.41, *P* <0.001). However, after controlling for parasite biomass, the relationship between VEGF and plasma lactate was no longer significant. There was no association between plasma VEGF and death (*P* = 0.93).

### Multivariable regression analysis

When combined in a logistic regression model with random effects for study year and inter-rater variability, a statistically significant independent association between microvascular obstruction, Ang-2 concentration and death could not be demonstrated (*P* = 0.06 for both, n = 126). The inclusion of an interaction term for microvascular obstruction and plasma Ang-2 concentrations was not significant (*P* = 0.88) and did not improve the model fit.

In a linear regression model with plasma lactate as the dependent variable, both microvascular obstruction and plasma Ang-2 concentrations were significant independent risk factors for increased plasma lactate (both *P* <0.001). In a model (n = 109) controlling for other factors that influence end organ perfusion, microvascular obstruction (*P* <0.001) and plasma Ang-2 concentration (*P* = 0.002) had a stronger correlation with plasma lactate than did estimated parasite biomass (*P* = 0.01), haemoglobin (*P* = 0.05), arterial oxygen saturation (*P* = 0.08), age (*P* = 0.09) or mean arterial blood pressure (*P* = 0.58).

## Discussion

This detailed clinical study provides further support for the belief that microvascular obstruction plays a fundamental role in the pathogenesis of severe falciparum malaria. Previous studies of patients with malaria have linked the degree of microvascular obstruction to disease severity [14, 15], but this larger series was also able to establish an association with the patients’ subsequent death; the demonstration of an *in vivo* association between microvascular obstruction and the presence of multi-organ dysfunction and acute kidney injury is also novel. The correlation between the OPS imaging results and the estimated total parasite burden suggests that the OPS imaging findings are representative of the sequestration that is present throughout the body in falciparum malaria and emphasises the potential utility of OPS imaging, given that it is able to assess a patient’s microcirculation during life. Plasma measures of endothelial activation and function were markedly deranged, with Ang-2 concentrations also correlating with the estimated parasite burden and outcome.

Plasma lactate, a measure of tissue hypoperfusion, was the strongest predictor of death in the series. As microvascular obstruction and endothelial activation are thought to lead to organ dysfunction through tissue hypoperfusion [7, 23], the finding that they were independently associated with plasma lactate is consistent with the hypothesis that they both have a causative role in the disease pathogenesis of the disease. The fact that microvascular obstruction and endothelial activation correlated more strongly with plasma lactate than indices such as blood pressure, oxygen saturation and haemoglobin highlights the pathological significance of the microcirculation in falciparum malaria.

OPS imaging has been used to assess the microcirculation in a variety of conditions [24–26]. Bacterial sepsis has been studied in the most detail and, as in falciparum malaria, measures of microvascular function are stronger predictors of outcome than a patient’s systemic hemodynamic status [15, 26]. However, there are clear differences in the OPS imaging findings between the two conditions. In patents with bacterial sepsis there is reduced capillary density and intermittent or absent perfusion in vessels of all sizes, changes which are reversible with the application of acetylcholine [26, 27]. In contrast, in severe falciparum malaria the striking finding is one of numerous persistently obstructed capillaries, an appearance which exactly replicates the histopathological findings at post-mortem examination [2–5]. These OPS findings also accord with the biochemical changes seen in the two conditions: in falciparum malaria elevated lactate: pyruvate ratios suggest anaerobic metabolism due to tissue hypoxia from an obstructed microcirculation rather than the accelerated aerobic glycolysis that is seen in sepsis [28, 29].

While the degree of visualized microvascular obstruction in the rectal mucosa correlated with many measures of disease severity, there was no association with coma depth. This presumably reflects the marked heterogeneity that is seen in the distribution of sequestration in different organs of the body [4], different parts of the brain [30, 31] and even in different vessels from the same area of the brain [31]. While the clinical presentation of cerebral malaria is correlated with the extent of cerebral sequestration on autopsy [4, 32, 33], the pathogenesis of cerebral malaria is generally believed to be more complex than simply resulting from direct hypoperfusion [31]; other factors including congestion and axonal injury are likely to contribute [32, 34]. The neuroanatomical location of pathological changes is also probably critical: the ascending reticular activating system controls consciousness and the extent to which these neurons are involved will determine coma depth [35].

Plasma Ang-2 concentrations were associated with disease severity and death as in other series of severe falciparum malaria [7, 9, 10] and studies of other critical illnesses [36, 37]. As with many biomarkers, it has been unclear whether elevations in Ang-2 are the cause or result of disease [38]. However, clinical studies of patients with bacterial sepsis have shown that elevations in plasma Ang-2 concentrations precede the appearance of end-organ injury and that serum from patients with sepsis disrupts the integrity of monolayers of endothelial cells *in vitro* via an Ang-2-dependent mechanism, suggesting that Ang-2 may be directly pathogenic [37]. Although Ang-2 has a variety of context-dependent effects on endothelial cells, by enhancing endothelial activation and increasing vascular permeability it is thought to play an important role in the pathogenesis of acute lung injury and multi-organ failure [36, 39], both of which are complications of falciparum malaria with a high attributable mortality [20]. Plasma Ang-2 concentrations were higher in the patients in this series with multi-organ dysfunction and while there was no clear association with acute lung injury, this was an infrequent finding in the series (six cases, only four of whom had an Ang-2 level available for analysis).

Nitric oxide is essential for the regulation of microvascular blood flow and inhibits the release of Ang-2 and other pro-inflammatory and vasoactive molecules [40, 41], however nitric oxide bioavailability is reduced in patients with falciparum malaria [6]. This is thought to result from hypoargininaemia [6], decreased nitric oxide synthase expression [42], quenching of nitric oxide by haemolysis-related cell-free haemoglobin [43] and impaired metabolism and clearance of the endogenous nitric oxide synthase inhibitor ADMA [44]. As endothelial nitric oxide concentrations decline, there is loss of vasodilatory tone which results in tissue hypoperfusion, lactic acidosis and organ dysfunction [8, 41]. Falling nitric oxide concentrations also lead to endothelial activation and the upregulation of endothelial receptors - including pRBC ligands like ICAM-1 - which facilitates pRBC cytoadherence [45]. This may cause further decreases in local nitric oxide concentrations, as the principal signal for nitric oxide production from the resting endothelium is the shear stress of flowing blood. Microvascular obstruction and loss of vasodilatory tone also lead to tissue hypoxia, another strong stimulus for endothelial activation [46].

It is clear to see how this might evolve into a vicious cycle, but in this series plasma Ang-2 concentrations did not correlate strongly with the visualized obstruction. This suggests that there is either a temporal dissociation between Ang-2 release and microvascular sequestration, that elevated Ang-2 concentrations are not the result of a simple, direct interaction between pRBCs and the endothelium, or that our statistical analysis is limited by the fact that we are comparing a local phenomenon (directly visualized microvascular appearances) with a systemic one (circulating plasma Ang-2 levels).

This study highlights the importance of microvascular obstruction in the unique presentation of falciparum malaria. Endothelial activation is present in many infectious and non-infectious conditions which lack falciparum malaria’s distinctive clinical and pathological findings [12, 13]. This includes vivax malaria [47, 48], a condition in which microvascular obstruction is largely absent [49] and organ failure and death less frequent [50]. The association between microvascular obstruction and the clinical manifestations of falciparum malaria emphasizes the relative clinical significance of the parasites in the RBCs that are obstructing the microcirculation when compared with circulating parasites which are represented by the peripheral parasitaemia [23]. For clinicians this means focusing primarily on a patient’s signs and symptoms and not being falsely reassured by a ‘low’ peripheral parasite count. When all patients with *P. falciparum* infection are considered, patients with higher peripheral parasite counts have a greater risk of complicated disease; however, in this series, where only patients with severe disease were enrolled, the median circulating parasite count was actually higher in survivors.

Plasma levels of VEGF were significantly decreased in this series and, as in previous studies, had an inverse correlation with disease severity [7]. This is in marked contrast to findings in murine models and argues against a role for excessive VEGF in pathogenesis of severe malaria. The inverse association between parasite biomass and VEGF is hypothesized to result from the absorption and accumulation of host VEGF within the parasitophorous vacuole by mature *P. falciparum* parasites, sequestered within the microvasculature [7]. VEGF stimulates the release of nitric oxide and upregulates the expression of nitric oxide synthase, thus the decreased VEGF concentrations provide another mechanism for decreased nitric oxide bioavailability. The significantly lower plasma Ang-2 concentrations in comatose patients replicates a similar finding in a previous Vietnamese study [9] and requires further evaluation.

## Conclusion

In conclusion, microvascular obstruction and systemic endothelial activation are both associated with tissue hypoperfusion, organ dysfunction and death in adults with falciparum malaria. These associations are consistent with the hypothesis that both processes play a significant role in the pathogenesis of the disease. Interventions which target either pathway may have therapeutic potential.

## Abbreviations

ADMA: Asymmetric dimethylarginine
AKI: Acute kidney injury
Ang-2: Angiopoietin-2
AOR: Adjusted odds ratio
CI: Confidence interval
GCS: Glasgow Coma Scale
IQR: Interquartile range
OPS: Orthogonal polarization spectral
*Pf* HRP2: *Plasmodium falciparum* histidine-rich protein 2
pRBC: Parasitized red blood cell
RBC: Red blood cell
RRT: Renal replacement therapy
VEGF: Vascular endothelial growth factor
WHO: World Health Organization

## References

[1] Marchiafava E, Bignami A (1894). On summer-autumnal fevers.

[2] Dudgeon LS, Clarke C (1917). A contribution to the microscopical histology of malaria. Lancet..

[3] Spitz S (1946). The pathology of acute falciparum malaria. Mil Surg..

[4] MacPherson GG, Warrell MJ, White NJ, Looareesuwan S, Warrell DA (1985). Human cerebral malaria. A quantitative ultrastructural analysis of parasitized erythrocyte sequestration. Am J Pathol.

[5] Turner GD, Morrison H, Jones M, Davis TM, Looareesuwan S, Buley ID (1994). An immunohistochemical study of the pathology of fatal malaria. Evidence for widespread endothelial activation and a potential role for intercellular adhesion molecule-1 in cerebral sequestration. Am J Pathol.

[6] Yeo TW, Lampah DA, Gitawati R, Tjitra E, Kenangalem E, McNeil YR (2007). Impaired nitric oxide bioavailability and L-arginine reversible endothelial dysfunction in adults with falciparum malaria. J Exp Med..

[7] Yeo TW, Lampah DA, Gitawati R, Tjitra E, Kenangalem E, Piera K (2008). Angiopoietin-2 is associated with decreased endothelial nitric oxide and poor clinical outcome in severe falciparum malaria. Proc Natl Acad Sci U S A..

[8] Yeo TW, Lampah DA, Kenangalem E, Tjitra E, Price RN, Anstey NM (2013). Impaired skeletal muscle microvascular function and increased skeletal muscle oxygen consumption in severe falciparum malaria. J Infect Dis..

[9] Prapansilp P, Medana I, Mai NT, Day NP, Phu NH, Yeo TW (2013). A clinicopathological correlation of the expression of the angiopoietin-Tie-2 receptor pathway in the brain of adults with Plasmodium falciparum malaria. Malar J..

[10] Conroy AL, Glover SJ, Hawkes M, Erdman LK, Seydel KB, Taylor TE (2012). Angiopoietin-2 levels are associated with retinopathy and predict mortality in Malawian children with cerebral malaria: a retrospective case-control study. Crit Care Med..

[11] Turner GD, Ly VC, Nguyen TH, Tran TH, Nguyen HP, Bethell D (1998). Systemic endothelial activation occurs in both mild and severe malaria. Correlating dermal microvascular endothelial cell phenotype and soluble cell adhesion molecules with disease severity. Am J Pathol.

[12] Lee WL, Liles WC (2011). Endothelial activation, dysfunction and permeability during severe infections. Curr Opin Hematol..

[13] Deanfield JE, Halcox JP, Rabelink TJ (2007). Endothelial function and dysfunction: testing and clinical relevance. Circulation..

[14] Dondorp AM, Ince C, Charunwatthana P, Hanson J, van Kuijen A, Faiz MA (2008). Direct in vivo assessment of microcirculatory dysfunction in severe falciparum malaria. J Infect Dis..

[15] Hanson J, Lam SW, Mahanta KC, Pattnaik R, Alam S, Mohanty S (2012). Relative contributions of macrovascular and microvascular dysfunction to disease severity in falciparum malaria. J Infect Dis..

[16] Maude RJ, Silamut K, Plewes K, Charunwatthana P, Ho M, Abul Faiz M (2014). Randomized controlled trial of levamisole hydrochloride as adjunctive therapy in severe falciparum malaria with high parasitemia. J Infect Dis..

[17] Maude RJ, Hoque G, Hasan MU, Sayeed A, Akter S, Samad R (2011). Timing of enteral feeding in cerebral malaria in resource-poor settings: a randomized trial. PLoS One..

[18] World Health Organization (2010). WHO guidelines for the treatment of malaria 2010.

[19] World Health Organization (2006). WHO guidelines for the treatment of malaria.

[20] Severe falciparum malaria. World Health Organization, Communicable Diseases Cluster. Trans R Soc Trop Med Hyg. 2000;94 Suppl 1:S1–90. 11103309

[21] Dondorp AM, Desakorn V, Pongtavornpinyo W, Sahassananda D, Silamut K, Chotivanich K (2005). Estimation of the total parasite biomass in acute falciparum malaria from plasma PfHRP2. PLoS Med..

[22] Jones CE, Darcy CJ, Woodberry T, Anstey NM, McNeil YR (2010). HPLC analysis of asymmetric dimethylarginine, symmetric dimethylarginine, homoarginine and arginine in small plasma volumes using a Gemini-NX column at high pH. J Chromatogr B Analyt Technol Biomed Life Sci..

[23] White NJ, Turner GD, Day NP, Dondorp AM (2013). Lethal malaria: Marchiafava and Bignami were right. J Infect Dis..

[24] Verdant C, De Backer D (2005). How monitoring of the microcirculation may help us at the bedside. Curr Opin Crit Care..

[25] De Backer D, Creteur J, Dubois MJ, Sakr Y, Vincent JL (2004). Microvascular alterations in patients with acute severe heart failure and cardiogenic shock. Am Heart J..

[26] De Backer D, Donadello K, Sakr Y, Ospina-Tascon G, Salgado D, Scolletta S (2013). Microcirculatory alterations in patients with severe sepsis: impact of time of assessment and relationship with outcome. Crit Care Med..

[27] De Backer D, Creteur J, Preiser JC, Dubois MJ, Vincent JL (2002). Microvascular blood flow is altered in patients with sepsis. Am J Respir Crit Care Med..

[28] Day NP, Phu NH, Mai NT, Chau TT, Loc PP, Chuong LV (2000). The pathophysiologic and prognostic significance of acidosis in severe adult malaria. Crit Care Med..

[29] James JH, Luchette FA, McCarter FD, Fischer JE (1999). Lactate is an unreliable indicator of tissue hypoxia in injury or sepsis. Lancet..

[30] Sein KK, Maeno Y, Thuc HV, Anh TK, Aikawa M (1993). Differential sequestration of parasitized erythrocytes in the cerebrum and cerebellum in human cerebral malaria. Am J Trop Med Hyg..

[31] Pongponratn E, Turner GD, Day NP, Phu NH, Simpson JA, Stepniewska K (2003). An ultrastructural study of the brain in fatal Plasmodium falciparum malaria. Am J Trop Med Hyg..

[32] Ponsford MJ, Medana IM, Prapansilp P, Hien TT, Lee SJ, Dondorp AM (2012). Sequestration and microvascular congestion are associated with coma in human cerebral malaria. J Infect Dis..

[33] Silamut K, Phu NH, Whitty C, Turner GD, Louwrier K, Mai NT (1999). A quantitative analysis of the microvascular sequestration of malaria parasites in the human brain. Am J Pathol..

[34] Medana IM, Day NP, Hien TT, Mai NT, Bethell D, Phu NH (2002). Axonal injury in cerebral malaria. Am J Pathol..

[35] Young GB (2009). Coma. Ann N Y Acad Sci.

[36] van Meurs M, Kumpers P, Ligtenberg JJ, Meertens JH, Molema G, Zijlstra JG (2009). Bench-to-bedside review: Angiopoietin signalling in critical illness - a future target?. Crit Care..

[37] David S, Mukherjee A, Ghosh CC, Yano M, Khankin EV, Wenger JB (2012). Angiopoietin-2 may contribute to multiple organ dysfunction and death in sepsis. Crit Care Med..

[38] Lee WL, Slutsky AS (2010). Sepsis and endothelial permeability. N Engl J Med..

[39] Agrawal A, Matthay MA, Kangelaris KN, Stein J, Chu JC, Imp BM (2013). Plasma angiopoietin-2 predicts the onset of acute lung injury in critically ill patients. Am J Respir Crit Care Med..

[40] Lowenstein CJ, Morrell CN, Yamakuchi M (2005). Regulation of Weibel-Palade body exocytosis. Trends Cardiovasc Med..

[41] Moncada S, Higgs A (1993). The L-arginine-nitric oxide pathway. N Engl J Med..

[42] Anstey NM, Weinberg JB, Hassanali MY, Mwaikambo ED, Manyenga D, Misukonis MA (1996). Nitric oxide in Tanzanian children with malaria: inverse relationship between malaria severity and nitric oxide production/nitric oxide synthase type 2 expression. J Exp Med..

[43] Yeo TW, Lampah DA, Tjitra E, Gitawati R, Kenangalem E, Piera K (2009). Relationship of cell-free hemoglobin to impaired endothelial nitric oxide bioavailability and perfusion in severe falciparum malaria. J Infect Dis..

[44] Yeo TW, Lampah DA, Tjitra E, Gitawati R, Darcy CJ, Jones C (2010). Increased asymmetric dimethylarginine in severe falciparum malaria: association with impaired nitric oxide bioavailability and fatal outcome. PLoS Pathog..

[45] Kim H, Higgins S, Liles WC, Kain KC (2011). Endothelial activation and dysregulation in malaria: a potential target for novel therapeutics. Curr Opin Hematol..

[46] Pober JS, Sessa WC (2007). Evolving functions of endothelial cells in inflammation. Nat Rev Immunol..

[47] Yeo TW, Lampah DA, Tjitra E, Piera K, Gitawati R, Kenangalem E (2010). Greater endothelial activation, Weibel-Palade body release and host inflammatory response to Plasmodium vivax, compared with Plasmodium falciparum: a prospective study in Papua, Indonesia. J Infect Dis..

[48] Barber BE, William T, Grigg MJ, Parameswaran U, Piera KA, Price RN (2015). Parasite biomass-related inflammation, endothelial activation, microvascular dysfunction and disease severity in vivax malaria. PLoS Pathog..

[49] Lacerda MV, Fragoso SC, Alecrim MG, Alexandre MA, Magalhaes BM, Siqueira AM (2012). Postmortem characterization of patients with clinical diagnosis of Plasmodium vivax malaria: to what extent does this parasite kill?. Clin Infect Dis..

[50] Severe malaria. Trop Med Int Health. 2014;19 Suppl 1:7–131. 10.1111/tmi.12313_2 25214480

